# The association between quality system development stage and the implementation of process-level patient safety themes in Dutch hospitals: an observational study

**DOI:** 10.1186/s12913-018-2997-x

**Published:** 2018-03-20

**Authors:** Steffie M. van Schoten, Janneke Hoogervorst-Schilp, Peter P. Groenewegen, Peter Spreeuwenberg, Cordula Wagner

**Affiliations:** 1 NIVEL – Netherlands Institute for Health Services Research, PO Box 1568, 3500 BN Utrecht, The Netherlands; 2 Department of Sociology, Department of Human Geography, Utrecht University, Utrecht, The Netherlands; 3 Department of Public and Occupational Health & EMGO Institute for Health and Care Research, Vrije Universiteit Medical Center (VUmc), Amsterdam, The Netherlands

**Keywords:** Quality, Healthcare, Quality improvement, Hospital quality systems, Patient safety

## Abstract

**Background:**

Quality systems are believed to be positively related to quality and safety in healthcare. However, there is no convincing evidence for this relationship in the literature. This study aims to examine the association between the development stage of hospital quality systems and the implementation of patient safety themes at the process level.

**Methods:**

This study combines data from a national survey on the development stage of quality systems in Dutch hospitals with results from an evaluation study of the Dutch Hospital Patient Safety Program. Data on the development stage of quality systems were collected in Dutch hospitals in 2011. A total of 73 quality coordinators completed a questionnaire (response rate 77%) covering five quality system domains: policy and strategy, human resource management, patient involvement, practice guidelines, and systematic quality improvement. Data were included on the implementation of five patient safety themes from the Dutch Hospital Safety Program. Process indicators for each theme were measured every four to six weeks, resulting in ten measurements in each hospital. Data were analyzed using multilevel analysis.

**Results:**

The mean score for hospital quality system development was 2.30 (range 1 to 3). The mean scores for the various quality system domains ranged from 1.56 (Patient Involvement) to 2.66 (Human Resources Management). The mean percentages for the implementation of the patient safety themes ranged from 12% for the ‘Pain’ process indicator to 73% for execution of the ‘Time-Out Procedure’. The intraclass correlation coefficients of the intercept-only model ranged between 11.6 and 51.6, which indicates large differences between hospitals in the implementation of the patient safety themes. Positive associations between quality system development stage and implementation of patient safety themes were found for four of the five patient safety themes, although they were not statistically significant.

**Conclusions:**

This study found no association between the development stage of a hospital quality system and the implementation of patient level safety themes at the process level. This rejects the hypothesis that quality improvement is caused by a positive relationship between structure (the quality system) and processes (the safety program implementation), which in their turn mold the quality of care at the patient level.

## Background

Growing concern about quality and safety within healthcare organizations made quality improvement an important topic in healthcare. According to Donabedian’s well-known model of quality improvement, quality can be achieved through a continuous cycle in which the organizational structure enhances organizational processes, which in turn leads to improved outcomes at the patient level [1–3]. In this model, the organizational structure is the quality system and is a prerequisite for continuous improvement [1–3]. A quality system is defined as a set of interacting activities, methods, and procedures aimed at directing, controlling, and improving the quality of care [4]. Quality systems contain quality improvement strategies in different areas such as policy, healthcare staff, patient involvement, and systematic measurement of outcomes [4–8]. The quality system is the structure within which quality improvement policies are embedded and this is hypothesized to have an influence on quality improvement activities at the process level. Ideally, in a developed quality system the quality activities are integrated into daily working processes throughout the healthcare organization. The policies and management of the healthcare organization ensure that this is done at the process level of organizations as well and this should become visible in a reduction of variation in processes of the healthcare organization and improvement of these processes over time. Most healthcare organizations have now implemented quality systems. However, there is large variation between countries and between individual healthcare organizations in terms of how well developed their quality system is [5, 7, 9, 10]. Insight into the development stage of a quality system is important, because it is assumed that this shapes the organizational processes, in turn leading to higher quality of care at the patient level [9, 11].

Several studies examined the relationship between quality systems or derivatives (such as hospital-level accreditation) and outcomes in terms of quality of care. Shaw et al. studied the effectiveness of different forms of external quality assessment of hospitals and found that accredited hospitals performed better on patient safety outcomes [11, 12]. Weiner et al. linked quality improvement with a set of patient safety indicators at the organizational level and found that higher percentages of physicians participating in quality improvement teams led to fewer postoperative complications and lower rates of technical difficulties with procedures [13, 14]. Kunkel et al. found higher scores for structure and outcomes when the implementation of a quality system was initiated by managers and when staff provided input to the quality system design. Subsequently, this was found to result in more advanced quality systems [15, 16]. Groene et al. found that better-developed quality systems were associated with lower rates of hospital complications and to some extent with fewer hospital readmissions in Spanish hospitals [9]. However, the same study found no association between the maturity of the quality system and hospital mortality and length of admission [9]. The European research project DUQUE assessed the association between quality management and patient outcomes in a wider setting: the European Union [17, 18]. Results from this project showed some associations between quality management measures at the hospital level and quality measures at the department level [19]. However, these associations were weak and the variability between countries was high [19].

Despite these examples from the literature, research into the relationship between quality systems and measures of quality and safety is limited and often restricted by small sample sizes and lack of availability of sufficient outcome measures [13, 14]. Although implementing quality systems in healthcare aims to improve the quality of care and patient safety by improving the processes, no clear evidence can be found in the literature that this is actually the case. This study aims to provide more insights into the association between the development stage of a hospital quality system (structure) and the quality of care at the process level. It should thereby be able to shed light on how quality systems work and provide more insights into the relationship between structure and processes in quality improvement. In this study, processes are quantified by the degree of implementation of patient safety themes within a national patient safety program. The degree of implementation of the patient safety themes is reflected in the scores for process indicators. The program will be described in more detail in the methods section. The research question addressed in this study is: *Is there a positive association between hospital quality system development stage and the implementation of patient safety themes on process level?* In line with Donabedian’s principles of quality improvement outlined above, we expect a positive association between the development stage of hospital quality systems and the implementation of patient safety themes. In other words, the patient safety themes measured in this study are expected to be more thoroughly implemented in hospitals with better-developed quality systems and this should be reflected in higher scores for the process indicators.

## Methods

### Study design

This study combines data from a national survey on the development stage of quality systems in hospitals (Study 1) with the results of an evaluation study of the Dutch Hospital Patient Safety Program (Study 2). The methods for each of the two studies are described below.

### Study 1. Structural level: Hospital quality system development

Since 1996, all Dutch healthcare institutions are obliged to have a quality system implemented in their organization. Therefore we chose to conduct this study with a more sophisticated measure namely the development stage of the quality system. Data on quality system development were collected during a large national survey on quality management in Dutch hospitals in 2011. All Dutch hospitals (*N* = 95) were approached and asked to participate in the study. The questionnaire was filled out by the quality coordinator of the hospital or a member of the management team. A total of 73 questionnaires were returned (response rate 77%). The questionnaire covered five domains of the hospital quality system: policy and strategy, human resource management, patient involvement, practice guidelines, and systematic quality improvement (see Wagner, 1999 for the items and psychometric properties of the questionnaire [4]). Data from this survey were used to assign each hospital to a development stage on a continuous scale from 0 to 3 for each of the five quality system domains, as well as the quality system overall. The development stage score reflects the level of implementation of the quality system in the organization. Stage 0 is ‘orientation and awareness’. In this stage, the organization has a notion that ‘something needs to be done’ about quality, but there are no systematic activities for quality assurance and improvement. In Stage 1 ‘preparation’, organizations create the necessary conditions for quality insurance and improvement. Stage 2 is ‘experimentation’ and involves developing quality improvement projects. Stage 3 is the highest stage of development and involves continuous improvement of quality of processes and outcomes, referred to as ‘integration into normal business processes’ [4–8, 18].

### Study.2 process level: Patient safety themes

The Dutch Hospital Patient Safety Program (hereinafter referred to as the Safety Program) was set up in 2008 to reduce preventable unintentional adverse events in Dutch hospitals by 50% by the end of 2012. The Safety Program consisted of ten patient safety themes to be implemented in the hospitals [20]. Clinical guidelines were developed by an expert group for each theme and presented in practical modules. Hospitals were given five years to implement these guidelines. Training and several practical recommendations for successful implementation of the protocol were offered to the hospitals. An evaluation study was performed between November 2011 and December 2012 to assess the extent to which six of the ten themes had been implemented. The study protocol was granted approval by the VU University Medical Center ethical review board in Amsterdam. This evaluation study took a representative sample of 38 hospitals (22 general, 12 tertiary teaching and 4 academic hospitals) from the total sample of Dutch hospitals, stratified by area and type of hospital. The participating hospitals were assigned to three of the themes in two groups. The hospitals were assigned to only one of the two clusters of three themes to limit the administrative burden for the hospital and increase the willingness to participate in the research. This resulted in a sample of 19 hospitals for every theme.

### Present study

**Fig. 1:**
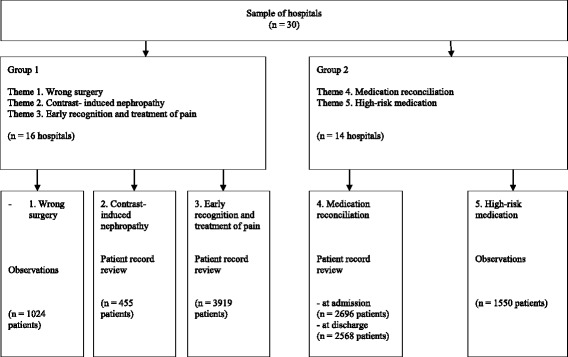
Data flow diagram for observations included in the present study

In the present study, data has been included for five of the ten safety themes. Of the five remaining themes, four themes were not part of the evaluation of the safety program: two of the safety themes were evaluated by means of existing national registration system data, two other themes were evaluated by means of another national research programme. The remaining six themes were part of the evaluation study of the safety program, but one theme was measured by means of qualitative data and therefore could not be included in the current research. This resulted in the inclusion of the five themes in the present study: (1) Wrong surgery, (2) Contrast- induced nephropathy, (3) Early recognition and treatment of pain, (4) Medication reconciliation at admission and discharge, and (5) High-risk medication. Table 1 gives an overview of the aims of the five different patient safety themes, the data collection, and the process indicators that were used as outcome measures in this study. Process indicators were measured every four to six weeks during a one-year follow-up for each of these five themes by a trained research assistant, resulting in a total of ten observation days for every theme in each hospital. The multiple measurements reduce the chance at a Type I error (false positive). A percentage was calculated for each process indicator, with higher percentages reflecting better implementation of the corresponding patient safety theme. Only hospitals that participated in both Study 1 and Study 2 were included in our study sample. This study therefore includes different numbers of hospitals and patients than the Safety Program. The data flow diagram for hospitals and patients included in the present study are shown in Fig. 1.

**Table 1:** Content and data collection of the five patient safety themes to evaluate implementation of the Safety Program

Patient Safety Theme	Aim within the Safety Program	Interventions in modules of the Safety Program	Design evaluation study	Process indicator evaluation study
Theme 1. Wrong surgery	Reducing the amount of wrong patient, wrong site, wrong procedure events. The aim is 0 events.	Time-out verification before surgery during which the total OR team is present and checks patient name, procedure to be performed and where to perform procedure (site and side).	Observational research with 6–10 observations of operations during 10 measurements in 18 hospitals.	Percentage of operations in which all 3 steps of the Time Out Procedure were performed correctly.
Theme 2. Contrast- induced nephropathy	Prevention of contrast-induced nephropathy by identifying all high-risk patients and taking suitable preventive measures	1. Identifying high risk patients (eGFR and medication review) 2. General prevention measures 3. Specific prevention measures	Patient record review with 20–25 randomly selected records during 10 measurements in 19 hospitals.	Percentage of high-risk patients who were hydrated before undergoing contrast administration.
Theme 3. Early recognition and treatment of pain	Reduce avoidable suffering by early recognition and treatment of pain.	1. three times a day: a standardized pain measurement 2. Register the pain scores 3. Take action at a pain score of 4 and higher	Patient record review with 20–25 randomly selected records during 10 measurements in 19 hospitals.	Percentage of postoperative patients who were in pain was measured in a standardized way three times a day in the first three days after surgery
Theme 4. Medication reconciliation	Medication reconciliation on admission and discharge.	Bundle 1. Medication reconciliation on admission 1. Obtain the primary medication history from the central pharmacy 2. Interview by a trained practitioner. 3. Develop a current and accurate medication review Bundle 2. Medication reconciliation at discharge 1. Develop a current and accurate medication review 2. Make an overview of discharge description authorized by the main specialist 3. At discharge review with the patient and/or responsible family member previous medication lists alongside the list of medication prescribed at discharge and reconcile the differences. 4. Communicate changes to a patients’ medication regimen to the pharmacist, general practitioner en other caregivers.	Patient record review with 20–25 randomly selected records during 10 measurements in 19 hospitals.	Percentage of patients for whom the bundle of medication reconciliation on admission and discharge had been implemented completely.
Theme 5. High-risk medication	Implementing the described process for preparing and administering parenteral medication	1. Process of preparing parenteral medication in non-acute situations 2. Process of administration of parenteral medication in non-acute situations (only this one was focus of the evaluation) 3. Process of preparing and administration in acute situations	Observational research with 20–25 observations of administration processes of parenteral medication at the intensive care unit, internal medicine and general surgery departments within during 10 measurements in 19 hospitals	Percentage of administration processes in which all recommended steps have been followed by the person administering the drug

Adopted from De Blok et al. 2013 [20]

### Data analyses

The mean scores for the development stage of the quality system were computed using descriptive analyses, as were the process indicators per patient safety theme. Separate multilevel logistic regression analyses were performed for every associations between hospital quality system development stage and the process indicators. We performed the analyses separately for each association and did not enter any additional variables due to limited power of the data. The data had a two-level structure, as the measurements at the patient level were clustered within hospitals. Intraclass correlation coefficients (ICC) were calculated to assess the degree to which variance in outcomes could be attributed to differences between hospitals. Patient characteristics were not included in the analyses. An ICC of > 20% is seen as substantial [21]. Descriptive analyses were performed using Stata version 11.1 and the multivariate analyses were performed using MLwiN version 2.24. Hypothesis testing was one-sided and *p*-values of < 0.10 were therefore considered to be statistically significant.

## Results

In total, data from 30 hospitals and 12,485 observations were included in this study. Table 2 shows the results of the quality system development stage variables and the results of the process indicators of the patient safety themes. The mean score for hospital quality system development stage was 2.30 (range 1 to 3). This indicates that all hospitals have implemented a quality system into their organization, and that most hospitals have designed quality improvement projects but don’t systematically use performance measures to adjust quality policy. The mean scores for the development stage for the different quality system domains ranged from 1.56 for Patient Involvement to 2.66 for Human Resources Management (range 1 to 3). This indicates that most hospitals have developed activities to train and educate healthcare staff in quality methods, but to a lesser extent involve patients in quality activities. The mean percentages for process indicators for the patient safety themes ranged from 12% for Pain indicator (standardized pain measurements, three times a day in the first three days after surgery) and Medication Reconciliation at Discharge to 73% for Time Out Procedure Execution (TOP). This indicates large differences between hospitals in the degree to which they have implemented the patient safety themes. Furthermore, the combination of the high SD and the broad range in scores reveal that most of the scores lie in both sides of the extremes. Analyses showed that this is being caused by differences between hospitals.

**Table 2:** Descriptors of hospital quality system development and patient safety themes

Hospital Quality System development	n	Mean	SD	Range
Overall	30	2.30	0.41	1–3
Policy and strategy	30	2.50	0.53	1–3
Human resource management	30	2.66	0.52	1–3
Patient involvement	30	1.56	0.92	0–3
Practice guidelines	30	2.54	0.60	1–3
Systematic quality improvement	30	2.22	0.93	1–3
Patient Safety themes		Mean %	SD	Range
Time out procedure	1024	73	44	0–100
- Check patient		- 96	- 21	- 0-100
- Check procedure		- 83	- 40	- 0-100
- Check side/site		- 92	- 27	- 0-100
- Focus during Time-out Procedure		- 55	- 50	- 0-100
Contrast- induced nephropathy	455	67	47	0–100
Pain process indicator 100%	3919	12	33	0–100
Medication reconciliation on admission	2696	35	48	0–100
Medication reconciliation at discharge	2568	12	33	0–100
High-Risk Medication	1550	18	38	0–100

Table 3 shows the variation between hospitals in the association between hospital quality system development stage and scores for process indicators for the patient safety themes. The wide range in scores for the various process indicators indicates large differences between hospitals in the implementation of the patient safety themes. For example, there was one hospital in our sample performing Medication Reconciliation on Admission only in 0.6% of admissions, whereas another hospital performed Medication Reconciliation in 96% of admissions. And for Contrast- Induced Nephropathy, the percentage of high-risk patients who were hydrated before undergoing contrast administration ranged between hospitals from 22% to 93%. The ICCs of the intercept-only model ranged between 23.1 for Contrast- Induced Nephropathy and 58.1 for Medication Reconciliation on Admission. The ICC indicates the percentage of the total variance in scores for the process indicators that came from the hospital level. For example, an ICC of 58.1 means that 58% of the total variance was related to differences between hospitals. The ICCs found in this study indicate that the differences between hospitals were relatively large in our sample. The ICC decreased when the hospital quality system development stage variable was added to the model, this decrease was statistically significant. In this model, the ICC ranged from 21.1 for Contrast-Induced Nephropathy to 58.1 for Medication Reconciliation on Admission. The decrease in ICC indicates that the differences between individual hospitals on the process indicator scores can be partly explained by differences in the development stage of the hospitals’ quality systems. The remaining variance can be explained by other differences between hospitals.

**Table 3:** Variation between hospitals in the association between hospital quality system development and outcomes for patient safety themes

	Intercept-only model		Quality system (overall)		1: Policy and strategy		2: Human resource management		3: Patient involvement		4: Practice guidelines		5: Systematic quality improvement	
Patient Safety themes	Hospital range	ICC	Hospital range	ICC	Hospital range	ICC	Hospital range	ICC	Hospital range	ICC	Hospital range	ICC	Hospital range	ICC
Time-out procedure	16–98	35.9	24–96	27.5	22–97	29.4	19–97	33.2	17–98	35.7	20–97	32.6	20–97	32.3
- Check patient	74–100	38.3	85–100	26.0	83–100	28.4	*	*	83-100	28.4	*	*	76-100	36.8
- Check procedure	16–100	51.6	23–100	46.5	20–100	49.0	18–100	50.2	17–100	51.9	18–100	50.7	22–100	47.0
- Check side/site	79–98	11.6	83–97	7.2	80–98	10.6	80–98	10.6	89–96	1.9	79–98	11.6	79–98	11.4
- Focus during TOP	18–88	19.8	19–87	18.4	18–88	19.0	18–88	19.8	20–87	17.2	18–89	20.1	18–88	20.1
Contrast- induced nephropathy	22–93	23.1	24–93	21.1	33–90	45.7	22–94	23.3	29–91	17.2	22–94	23.4	22–93	22.7
Pain process indicator 100%	0.4–56	38.9	0.4–55	39.1	0.4–54	38.4	0.4–54	38.9	0.4–52	37.1	0.4–53	37.5	0.4–55	39.0
Medication reconciliation on admission	0.6–96	58.1	0.6–0.96	58.4	0.6–96	58.3	0.5–97	59.4	0.7–95.7	55.9	0.5–97	59.0	0.5–96.8	59.9
Medication reconciliation at discharge	0.1–61	50.7	0.1–56.9	49.1	0.2–47	43.2	0.1–57	49.4	0.1–59	50.9	0.1–52	46.2	0.1–58	49.9
High-Risk Medication	0.8–69	38.9	0.8–68	38.3	0.7–69	39.2	0.9–65	35.9	1.2–60	31.3	0.7–68	38.8	0.7–69	38.9

*there were not enough observations to calculate the associations between Human Resource Management & Practice Guidelines and Check Patient

Table 4 shows the association between hospital quality system development stage and scores for the process indicators for the patient safety themes. For four of the five safety themes, positive associations were found between the overall quality system development stage and the scores for the process indicators. However, none of these associations were statistically significant. Inconsistent results were found when examining the associations between the different dimensions of the quality system development stage. Some of the associations were positive and some were negative, but only a few were statistically significant. Statistically significant positive associations were found between the Policy and Strategy dimension and the patient safety themes Medication Reconciliation at Discharge and Contrast- Induced Nephropathy. Statistically significant positive associations were found between Patient Involvement and the patient safety themes Contrast- Induced Renal Failure and High-Risk Medication. One additional analysis was performed, as the association between the hospital quality system development stage and the process indicators for the patient safety theme Wrong Surgery was in the opposite direction to what had been hypothesized. This association was investigated in more detail by examining the association between the hospital quality system development stage and the three individual checks of the TOP. Negative associations were found between the development stage of the quality system and the checks on the patient identity and the check on the side/site.

**Table 4:** Separate associations between hospital quality system development and outcomes for patient safety themes

	Model 1 Quality system (overall)		1: Policy and strategy		2: Human resource management		3: Patient involvement		4: Practice guidelines		5: Systematic quality improvement	
Outcome	Estimate (SE)	R^2^	Estimate (SE)	R^2^	Estimate (SE)	R^2^	Estimate (SE)	R^2^	Estimate (SE)	R^2^	Estimate (SE)	R^2^
Time-out procedure	−1.89 (0.82)	32.3	− 1.33 (0.66)	25.4	− 1.25 (0.89)	11.4	−0.16 (0.38)	0.9	−1.08 (0.74)	13.7	−0.57 (0.37)	14.7
- Check patient	−2.49 (1.13)	43.5	−1.65 (0.88)	36.2	–	–	−0.73 (0.40)	36.0	–	–	0.19 (0.51)	6.1
- Check procedure	−2.61 (1.37)	18.3	−1.46 (1.06)	9.6	−1.78 (1.48)	5.2	−0.18 (0.58)	−1.2	−1.02 (1.13)	3.3	−1.02 (0.55)	16.8
- Check side/site	−0.96 (0.49)	40.8	−0.32 (0.44)	8.8	−0.40 (0.54)	8.8	−0.59 (0.14)	8.5	0.09 (0.46)	−0.5	−0.02 (0.23)	1.0
- Focus during TOP	−0.75 (0.62)	8.8	−0.45 (0.50)	4.8	−0.28 (0.61)	−0.1	− 0.39 (0.24)	15.8	− 0.08 (0.53)	−1.9	−0.02 (0.27)	−1.7
Contrast- induced nephropathy	0.86 (0.68)	0.11	0.29 (0.44)**	14.0	−0.08 (0.63)	−0.9	0.55 (0.25)*	30.8	−0.11 (0.45)	−1.7	−0.17 (0.29)	2.2
Pain process indicator 100%	0.09 (0.98)	−0.5	−0.55 (0.75)	2.3	0.41 (0.89)	0.1	0.28 (0.37)	7.3	−0.66 (0.62)	5.8	0.12 (0.40)	−0.2
Medication reconciliation on admission	0.64 (1.40)	−1.3	0.53 (0.99)	−0.9	0.42 (1.06)	−5.5	0.97 (0.76)	8.4	−0.41 (1.00)	−3.8	−0.13 (0.67)	−7.5
Medication reconciliation at discharge	1.01 (1.30)	6.3	1.73 (0.87)*	26.0	−0.64 (0.87)	5.0	−0.002 (0.74)	−0.8	1.33 (0.92)	16.6	0.21 (0.59)	3.1
High-Risk Medication	0.51 (0.91)	2.4	−0.04 (0.68)	−1.2	−0.81 (0.61)	12.0	0.89 (0.42)*	28.4	0.20 (0.66)	0.1	0.20 (0.45)	−0.1

* *p* < 0.10, **p < 0.05

## Discussion

Quality systems are hypothesized to have an influence on quality improvement activities at the process level, which in turn influences hospital outcomes at the patient level. This study linked the development stage of a hospital quality system (structure) to the implementation of patient safety themes at the process level (processes) in Dutch hospitals. This was measured by means of process indicators. We found no statistically significant associations between the development stage of a quality system and the implementation of patient safety themes. Some statistically significant associations were found between dimensions of the quality system development stage and process indicators for the implementation of patient safety themes. However, given the large number of associations that were tested compared to the limited number of statistically significant associations found, it is possible that these findings can be attributed to chance. We therefore conclude that this study found no conclusive evidence for a positive association between the development stage of a hospital quality system and the implementation of patient safety themes at the process level. An interesting finding is the large variation in scores of hospitals on the implementation of the patient safety themes.

The results of this study could indicate that no association exists between structure and processes in the cycle of quality improvement. However, other explanations seem more likely. These might be associated with the translation of the quality systems into quality improvement activities at the process level on the one hand, or with the complexity of implementation itself on the other. Firstly, several factors could hinder the translation of structural factors into more practical activities that can be applied on the work floor. For example, the extent to which hospital management uses a top-down approach to make a connection between the quality system and processes in their hospital could influence the extent to which quality system components are adequately translated into quality activities at the hospital department level. Alternatively, the attitudes of healthcare staff to quality improvement might play a role in the extent to which parts of the quality system are adopted and adhered to at the clinical level These factors might mediate the relationship between quality systems and processes, making it complex to measure and attribute to the variation of scores on process indicators that we found in this study. Future research into the attitudes of healthcare staff towards quality procedures and the extent to which these are applicable and adhered to in daily practice could provide insights in the context in which an association between a quality system and process indicators is being established.

Secondly, the structure of quality management is unlikely to be the only explanatory factor in the degree of implementation of patient safety themes. Implementation is seen as highly complex, as the extensive body of literature on barriers and facilitators for implementation illustrates [22–25]. Besides organizational aspects that can facilitate implementation of interventions into healthcare practice, individual factors play an important part as well. More specifically, implementation is assumed to require behavioral changes by individual healthcare staff and numerous theories on behavioral change have been developed to guide interventions. Recently, the theoretical domains framework has captured the key concepts of all these different theories in a comprehensive framework of 14 domains that can help to explain implementation problems and provide input for the design of interventions [26, 27]. The theoretical domains framework highlights not only the importance of a theoretical grounding in the design of interventions but also the broad spectrum of concepts that are associated with healthcare staff behavior [26, 27]. These behavioral components were not measured in the present study but might have been important in the level of implementation of the patient safety themes and might explain the large differences that between hospitals.

Lastly, it can be argued that the indicators used in this study cannot be seen as a representative set of indicators to define an association between the quality system development and process indicators since there is no direct match between the indicators of the quality system and the process measures. However, according to the idea of quality improvement a quality system sets the preconditions for the organization to improve quality by integrating quality improvement policies in daily working processes. This would still lead to the expectation that the quality system has a general effect on outcomes at process level even if there is no direct link between the indicators. Related to this issue, the process indicators that were used in this study were part of a national safety programme that was designed separately from the existing quality system of the hospitals. Therefore these indicators are possibly less integrated in the hospital organization, making it difficult to find an association between the hospital quality system and the chosen process indicators. In future research, process indicators with a more direct link to hospital quality systems could be considered.

### Strengths and limitations

This study expands upon existing literature on the effectiveness of quality systems by studying the association between organizational structure and organizational processes. This corresponds to current views that quality systems do not directly influence outcomes at the patient level, but that this is achieved through the improvement of processes [9, 11, 19, 28]. Taking all the different dimensions of a quality system into account lets our study offer a broad picture of the associations between structure and processes within the theory of quality improvement. The multilevel approach that was used in the present study accounted for the clustering of observations at the patient level within hospitals, allowing data at the hospital level to be linked to data at the patient level.

We acknowledge several limitations to our study. Firstly, the independent variable in our study (quality system development) relied on self-reported data and this might have led to socially desirable responses. The measurement instrument used for determining the development stage of hospital quality systems has been widely used and validated [4–8]. We are therefore confident that we have captured the key aspects of the development of hospitals’ quality systems. Secondly, this study combined data from two different studies. Only the hospitals that participated in both data collections could therefore be included in the present study, which limited the number of hospitals. Thirdly, the variation of scores for our independent variable (the quality system development stage) was small. This may have limited the possibilities for finding significant associations. Future research should explore or develop new and more sensitive possibilities for measuring (aspects of) quality systems. And lastly, every participating hospital was assigned to only one of the two clusters of three themes. Ideally, data for all the safety themes would be available for every participating hospital. However, the hospitals were randomly assigned to one of the two clusters of themes and therefore we do not expect that this approach biased the results of this study.

## Conclusions

This study found no association between the development stage of a hospital quality system and the degree of implementation of patient level safety themes at the process level. This rejects the hypothesis that quality improvement is caused by a positive association between structure and processes, which in turn contribute to outcomes at the patient level. Several factors may have contributed to the results of this study. Future research should try to resolve methodological constraints associated with the measurement of quality systems as well as quantifying more factors associated with the implementation of quality improvement interventions.
